# Risk of childhood acute lymphoblastic leukaemia following parental occupational exposure to extremely low frequency electromagnetic fields

**DOI:** 10.1038/bjc.2011.365

**Published:** 2011-09-13

**Authors:** A Reid, D C Glass, H D Bailey, E Milne, N H de Klerk, P Downie, L Fritschi

**Affiliations:** 1 Epidemiology Group, Western Australian Institute for Medical Research, M519, University of Western Australia, 35 Stirling Highway, Crawley, Western Australia 6009, Australia; 2 Monash Centre for Occupational and Environmental Epidemiology, Monash University, Melbourne, Victoria, Australia; 3 Telethon Institute for Child Health Research, University of Western Australia, Perth, Western Australia, Australia; 4 Department of Paediatrics, Monash University, Melbourne, Victoria, Australia; 5 Department of Paediatric Haematology-Oncology, Monash Children's Hospital, Melbourne, Victoria, Australia

**Keywords:** acute lymphoblastic leukaemia, parental occupational exposure, electromagnetic fields

## Abstract

**Background:**

Earlier studies have reported moderate increases in the risk of acute lymphoblastic leukaemia (ALL) among children whose mothers have been occupationally exposed to extremely low frequency (ELF) electromagnetic fields. Other studies examining parental occupational exposure to ELF and ALL have reported mixed results.

**Methods:**

In an Australian case–control study of ALL in children aged <15 years, parents were asked about tasks they undertook in each job. Exposure variables were created for any occupational exposure before the birth of the child, in jobs 2 years before birth, in jobs 1 year before birth and up to 1 year after birth.

**Results:**

In all, 379 case and 854 control mothers and 328 case and 748 control fathers completed an occupational history. Exposure to ELF in all time periods was similar in case and control mothers. There was no difference in exposure between case and control fathers. There was no association between maternal (odds ratio (OR)=0.96; 95% CI=0.74–1.25) or paternal (OR=0.78; 95% CI=0.56–1.09) exposure to ELF any time before the birth and risk of childhood ALL.

**Conclusion:**

We did not find an increased risk of ALL in offspring of parents with occupational exposure to ELF.

Non-ionising radiation comprises oscillating electric and magnetic field energy waves that travel at the speed of light. At the extremely low frequency (ELF) end of this spectrum, it is the separate electric and magnetic fields that are of interest, and it is usually considered that radiation effects only become important above 3 kHz (International Agency for Research on Cancer, 2002). Such ELF fields are produced by powerlines and electrical wiring as well as the motors and heating coils of electrical equipment and appliances.

Several pooled analyses and reviews have found a generally consistent, albeit moderate, association between exposure of the child to electromagnetic fields and the risk of childhood leukaemia (Ahlbom *et al*, 2000; Greenland *et al*, 2000; Kheifets *et al*, 2010), although no definitive biological mechanism has been identified (Schuz and Ahlbom, 2008).

Fewer studies have examined the association between parental occupational exposure to ELF fields and risk of acute lymphoblastic leukaemia (ALL) in their offspring. One case–control study has reported an increased risk of childhood ALL (RR=7.0; 95% CI=1.59–30.79) in the children of mothers who worked at home during their pregnancy. The majority of these women sewed fabric using a machine (Infante-Rivard *et al*, 1991) from which they were exposed to ELF magnetic fields (Infante-Rivard, 1995). Another case–control study specifically examined ELF occupational exposure in mothers during pregnancy and found a moderately increased risk for ALL in their offspring. The highest risk (OR=2.5; 95% CI=1.2–5.0) was reported among mothers with a maximum occupational exposure of ⩾0.4 *μ*T in a given occupation (Infante-Rivard and Deadman, 2003). This study used the expert assessment method for assessing exposure, in which an expert assigns exposure based on the tasks performed in each job (Siemiatycki *et al*, 1981).

Other studies using less accurate exposure assessment methods have examined the relationship between parental occupational exposure to magnetic fields and risk of ALL in the offspring with inconsistent findings. Studies that inferred exposure to ELF fields from the job title (Sorahan *et al*, 1999) or by relating job title to a job exposure matrix (Hug *et al*, 2010) generally did not find an association with either maternal or paternal exposure. However, McKinney *et al* (1991) found an increased risk in the offspring of mothers who worked in catering, cleaning and hairdressing and food-related occupations in the 40 weeks before conception although no specific range of occupations accounted for the increased risk. Similarly, Feychting *et al* (2000) report a two-fold increased risk for leukaemia in the children of men occupationally exposed to magnetic fields ⩾30 *μ*T in the period 2–26 weeks before the birth of the child. Exposure was determined by relating job title to a job exposure matrix.

This study examines the risk of ALL in the children of parents with preconceptional, periconceptional, gestational and post-natal occupational exposure ELF fields determined by the expert assessment method.

## Materials and methods

### Study population

Aus-ALL was a population-based case–control study of ALL in children aged <15 years in Australia. Full details of the study population and recruitment methods used have been published elsewhere (Milne *et al*, 2009; Bailey *et al*, 2010). Briefly, cases and their families were identified and recruited from 10 paediatric oncology centres in Australia and were eligible if they were diagnosed between 1 July 2003 and 31 December 2006 and were in remission. Three controls per case were recruited in seven recruitment waves over the same period, by random digit dialling (RDD) and were frequency matched to cases on age, sex and State of residence. The biological mother had to have sufficient English language skills to complete the questionnaires for any case and control child to be eligible to participate in the study. Human Research Ethics Committee approval was obtained from all participating hospitals.

### Data collection

A written questionnaire seeking information on age, smoking history, residential postcode, ethnicity, alcohol intake, birth order, maternal age at child's birth, highest level of education, family income and whether child had a birth defect was completed by a parent. In addition, a lifetime occupational history up to the birth of the child was completed for both parents and up to 1 year after the birth of the child for mothers. Information provided for each job included the year started and finished, the job title, the employer, main tasks and number of hours worked each week. An initial review of the literature regarding parental occupational exposures and childhood ALL identified a number of hypotheses to test including exposure to ELF. We then identified those jobs, which had potential for exposure to any of these agents and adapted existing questionnaires for each of these jobs (as listed in the Materials and methods). Each occupational history was examined manually to identify any of these jobs or similar jobs (e.g., barber/hairdresser/beautician) and the appropriate questionnaire was assigned.

Trained interviewers telephoned and asked parents further specific questions about their job if they reported ever working as a; barber, carpenter, chemist, office worker, drycleaner, driver, engineering technician, farmer, health professional, storeman, fisherman, petrol station attendant, labourer, mechanic, miner, metal worker, painter, printer, radio operator, railway worker, shoemaker or teacher or if they worked in particular industries; aluminium, forestry, military, leather, oil refining, rubber and textile. An expert (DG) reviewed the job histories and the answers to the job-specific questions to assess exposure to ELF. She was blinded as to case status and determined the likelihood of exposure as no exposure, possible exposure or probable exposure and the level of that exposure as high, medium or low. The number of years in the job (duration of exposure) was taken from the job history.

Interviewers also asked whether parents used or worked near electrical equipment of different sizes, that is, *small* hand-held equipment, *medium-sized* equipment such as a household washer or dryer or *large* equipment such as heavy duty industrial equipment larger than a household washer or dryer. Parents were allocated high exposure if they worked within 1 m of large equipment, medium exposure if they worked 1 m from large equipment or within 1 m of medium-sized equipment or and low exposure if they reported any other electrical exposure. Welders were assigned high exposure for the time spent welding.

### Creation of exposure variables

Exposure variables were created for specific time periods and for specific jobs in order to capture the exposure that occurred during the periods of preconception and periconception, gestation and post-natal. The specific time periods covered were before and up to the child's birth and after the birth of the child (mothers only). The specific jobs covered (1) exposure in the job held 2 years before the birth of child (mothers only – preconception and periconception), (2) exposure in the job held 1 year before the birth of child (mothers – gestation and fathers – periconception) and (3) 1 year after the birth (mothers only – post-natal). Job held up to 2 years before the birth identifies the job that was held for the majority of the time between the birth of the child to a maximum of 2 years before the birth of the child. Duration of exposure contributes to the exposure variable by helping to identify the job that was held for the longest time in the period up to 2 years before the birth of the child. Similarly, the job held up to 1 year before the birth of the child identifies the job that was held between the birth of the child and up to 1 year before the birth of the child.

Exposures for each time period were coded as none (no exposure) low (probable low exposure and possible low/medium/high exposure) and medium/high (probable medium or high exposure). Exposure variables in specific jobs up to 2 years and 1 year before the birth of the child were created by identifying the exposures in the job that was closest to the specified time period. Because of smaller numbers, these variables were recoded to not exposed and exposed.

An area-based measure published by the Australian Bureau of Statistics, the Index of Relative Socioeconomic Disadvantage (IRSD) and allocated according to the residential address at the time of entry into the study was used to assess socioeconomic status. Further details of this process have been published elsewhere (Bailey *et al*, 2010).

### Statistics

Frequencies of demographic and exposure variables were compared between cases and controls using *χ*^2^-tests and Fisher's exact tests. Maternal and paternal estimates of exposure were related to the risk of ALL in their offspring using logistic regression and adjusting for potential confounders. Maternal models were adjusted for the child's sex and age at diagnosis, socioeconomic status, maternal smoking and drinking during pregnancy and maternal age at the time of birth. Paternal models were adjusted for the child's sex and age at diagnosis, socioeconomic status, paternal smoking during the birth year and paternal drinking alcohol 1 year before pregnancy. Models examined the relationship between occupational exposure at anytime prior and up to the birth of the child, in the job 2 years before the birth of the child, in the job 1 year before the birth of the child and in the period up to 1 year after the birth of the child. The level of statistical significance was set at *P*<0.05 and all analysis was undertaken using Stata 10.1 (StataCorp, 2007).

## Results

We were notified of 568 incidence cases of ALL. Forty-nine were ineligible to participate: 30 were from non-English speaking backgrounds, 12 were overseas visitors, the biological mother was unavailable for three cases and four cases did not reach remission. Parents of 416 (80%) of 519 eligible cases consented to participate in the study. An occupational history was obtained from 379 mothers (73%) and 328 fathers (63%). Two thousand nine hundred and forty-seven eligible control families were identified through RDD and 2071 (70%) agreed to participate. We only recruited 1361 of these families to the study because of age and sex frequency-matching quotas. Of those who were recruited, 854 (63%) mothers and 748 (55%) fathers provided an occupational history.

Case mothers and fathers were slightly younger and drank less alcohol than control mothers and fathers. Case fathers were more likely to smoke cigarettes during pregnancy than control fathers. There was no difference in socioeconomic disadvantage as measured by the IRSD of residence between case and control parents (Table 1).

The distribution of exposure to ELF in all time periods was similar between the case and control mothers, except for mothers who had exposure in their job up to 1 year after the birth. More case mothers had low exposure compared with control mothers and more control mothers had moderate or high exposure compared with case mothers (*P*=0.052) (Table 2). There was no difference in the distribution of exposure to ELF between case and control fathers in either of the periods examined.

There were no associations between parental occupational exposure to ELF and risk of childhood ALL for any of the time periods we examined (Table 3).

## Discussion

This was a national population-based case–control study of childhood ALL conducted in Australia: it used the best method available to minimise bias in its ascertainment of occupational exposure. We did not find any association between parental occupational exposure to ELF and risk of ALL in the child.

Our findings do not support those of an earlier case–control study conducted in Canada, which used a method similar to ours to ascertain occupational exposure (Infante-Rivard and Deadman, 2003). That study of 491 age- and sex-matched cases and controls reported an increased risk of ALL in the offspring of mothers whose exposure level was in the highest 10% for: cumulative exposure, OR=1.6 (95% CI=1.0–2.5), and average exposure, OR=1.4 (95% CI=1.0–2.2). The highest odds ratio (OR=2.5; 95% CI=1.2–5.0) was found among women with an exposure level ⩾0.4 *μ*T. In contrast, we report an OR=0.96 (95% CI=0.74–1.25) among children whose mothers were exposed to moderate/high levels of ELF at any time before the child's birth. However, our ORs attenuated to near unity when occupational exposure to ELF was examined specifically in the job up to 2 years before the birth of the child (OR=1.13; 95% CI=0.87–1.48), and in the job up to 1 year before the birth of the child (OR=1.11; 95% CI=0.86–1.44). Our estimates may be lower than those reported by the Canadian Study because in that study mothers with the highest 10% of exposure were compared with the other 90%, while we compared those who had low or moderate/high exposure with those who were not exposed. Also, the Canadian Study used a cumulative measure of exposure while we examined occupational exposure at anytime before the birth of the child or in the job 2 years and 1 year before the birth of the child, which captured exposure during specific periods rather than cumulative exposure.

Other studies have examined parental occupational exposure to ELF and reported an increased risk of ALL in their offspring. Pearce *et al* (2007) found an increased risk for lymphoid leukaemia among the offspring of fathers who were electricians (OR=1.59; 95% CI=1.12–2.26). In that large registry-based case–control study of 744 cases and 30 947 registry-based controls and 70 800 Cumbrian births database controls, occupation was taken from the child's birth certificate and likely exposure to ELF was identified from the job title. Similarly, Smulevich *et al* (1999) reported an excess risk for ALL in the offspring of mothers (OR=5.2; 95% CI=1.6–16.8) and fathers (OR=4.6; 95% CI=1.8–11.9) occupationally exposed to ELF at any time before the conception of the child. Exposure was determined by job title and information provided by the parents was related to a job exposure matrix by an occupational hygienist. Feychting *et al* (2000) reported an increased risk for all leukaemia types in the offspring of fathers occupationally exposed to ELF in a Swedish registry-based cohort study (RR=2.0; 95% CI=1.1–3.5). Job title from the previous census was linked to a job exposure matrix to ascertain exposure. The difficulty with determining exposure from job title or relating job title to a job exposure matrix is that people with the same job name may have very different work conditions but be classified incorrectly as having the same exposure (Semple *et al*, 2004). Similarly, for studies that specifically asked the parents about their exposures, recall may have been different among case and control parents. In all of these studies (except Smulevich *et al*), the ORs were not highly elevated, suggesting that if there is a causal association with ALL, it does not explain a large proportion of the risk.

In contrast, other studies have examined the risk of ALL in offspring following parental occupational exposure to non-ionising radiation and found no excess risk. A case–control study of 1461 cases from the United Kingdom determined occupational exposure to a range of substances by an expert panel that related job title and industry to epidemiological literature, job descriptions and monitoring data to create occupational groups that were considered homogenous to specific exposures (McKinney *et al*, 2003). Mother's exposure to ELF in the periconceptional period (OR=0.85; 95% CI=0.66–1.10) or father (OR=1.16; 95% CI=0.93–1.50) was not associated with an increased risk of ALL in the offspring. Similarly, Hug *et al* (2010) found no increased risk for all leukaemia types in the offspring if mothers (OR=0.89; 95% CI=0.65–1.23) or fathers (OR=0.85; 95% CI=0.7–1.03) were exposed to ELF >0.2 *μ*T during the periconceptional period in their large German case–control study of 846 cases and 2382 controls. In that study, exposure was derived by linking job title to a job exposure matrix. Likewise, Sorahan *et al* (1999) reporting on 15 276 matched childhood cancer cases and controls (6610 leukaemias) from a large English national case–control study found no excess risk for all childhood leukaemia types in mothers who were exposed to ELF before, during or after pregnancy. Again, that study derived exposure from job title. Similar to those studies mentioned above, the exposure assessments in these studies may have been misclassified in the process of deriving exposure from a job exposure matrix or job title.

We used a well-recognised method for assessing occupational exposure in a population-based study (McGuire *et al*, 1998). More information was sought about specific jobs in which exposure to ELF may have occurred. This information was then assessed by an occupational hygienist who determined if the parent had been exposed to ELF, and the level and duration of that exposure. The exposure determination was largely related to questions about proximity to electrical machinery, rather than soliciting more general information about the tasks carried out. The participants also answered a number of questions about the tasks affecting several other occupational exposures including solvents, paint, resins, ionising radiation, lead and exhaust exposure. Under these circumstances, recall bias could have had an effect; however, this method is less likely to be hampered by recall bias (than are those studies that rely on self-reported exposure to a single agent) or misclassification (as can occur when a job exposure matrix is used). If recall bias had played a significant role in this study, our results would have been more likely to have shown a positive association.

The main limitation of this study is that different proportions of cases and control parents returned questionnaires and completed telephone surveys. At the time the study was conducted, over 95% of Australian households had a landline telephone, so were eligible to participate as controls. There is a possibility of selection bias despite adjusting our effect measures for various factors that might be associated with participation. The response from control mothers and fathers to completing the occupational histories specifically was moderate with mothers at 63% and fathers 55%. Case mothers responded reasonably well (73%) but case fathers less so (63%). In order to understand the possible impact of selection bias on our results, we examined the prevalence of occupational exposure to non-ionising radiation among the deciles of socioeconomic disadvantage. For exposure in specific jobs 1 and 2 years before pregnancy, those with higher socioeconomic status had higher occupational exposure to ELF. This would probably result in an underestimate of the effect of exposure to ELF and risk of childhood leukaemia in this study because we recruited more higher socioeconomic status controls and cases than lower socioeconomic controls and cases. There was no difference by socioeconomic status in all ELF exposure before pregnancy or exposure post-pregnancy. There may be some selection bias among participants overall, but this should not have impacted on the exposure information collected. Job title and tasks were collected rather than participants being directly asked about their exposure to electrical fields. Strengths of this study include the large numbers of cases and controls and the high prevalence of exposure to non-ionising radiation so that we were able to examine exposure at different times throughout the pregnancy. Selection bias related to high SES among controls is not confined to studies using RDD; it has also been seen in studies involving sampling from provincial health insurance records (Mezei and Kheifets, 2006) using files of residents (Richiardi *et al*, 2002) or primary health-care registers (Law *et al*, 2002; Mensah *et al*, 2007) and household doorknocking (Brogan *et al*, 2001).

In conclusion, we did not find an increased risk of ALL in the offspring of parents with occupational exposure to ELF.

## Figures and Tables

**Table 1:** Parental characteristics by case or control status

	**Mother**		**Father**	
	**Case**	**Control**	**Case**	**Control**
	***N*=379**	***N*=854**	***N*=328**	***N*=748**
	***N* (%)**	***N* (%)**	***N* (%)**	***N* (%)**
*Age at birth* a ^,^^*^				
<25 years	56 (15)	90 (11)	28 (9)	52 (7)
25–34 years	259 (68)	569 (67)	213 (65)	471 (63)
35+ years	64 (17)	195 (23)	87 (27)	225 (30)
				
*Drank alcohol during pregnancy* a ^,^^*^				
No	264 (70)	505 (59)	47 (14)	75 (10)
Yes	115 (30)	349 (41)	281 (86)	673 (90)
				
*Smoked during pregnancy*				
No	301 (79)	712 (83)	214 (65)^*^	544 (73)
Yes 1–14	43 (11)	74 (9)	35 (11)	78 (10)
Yes 15+	35 (9)	68 (8)	79 (24)	126 (17)
				
*Index of relative socioeconomic disadvantage decile* b				
1	15 (4)	47 (6)	11 (3)	34 (5)
2	27 (7)	49 (6)	23 (7)	39 (5)
3	34 (9)	54 (6)	23 (7)	54 (7)
4	33 (9)	66 (8)	28 (9)	59 (8)
5	34 (9)	82 (10)	29 (10)	65 (9)
6	42 (11)	91 (11)	37 (11)	80 (11)
7	36 (10)	114 (13)	33 (10)	95 (13)
8	44 (12)	120 (14)	40 (12)	106 (14)
9	55 (15)	97 (11)	54 (17)	88 (12)
10	57 (15)	127 (15)	48 (15)	121 (16)

a Mother's age at birth, father's age 1 year before birth; mother drank alcohol during pregnancy, father drank alcohol 1 year before pregnancy.

b The ABS published deciles of the Index of Relative Socioeconomic Disadvantage of the 2006 Census Collection District of the address at the time of entry to the study 1=most disadvantaged 10%, 10=most advantaged 10%.

^*^P-value <0.05.

**Table 2:** Parental occupational exposure to ELF by case or control status

	**Mothers**			**Fathers**		
**Exposure time period**	**Case, *N* (%)**	**Controls, *N* (%)**	***P*-value**	**Case, *N* (%)**	**Controls, *N* (%)**	***P*-value**
*Exposure anytime before birth*						
No/low	123 (32)	257 (30)	0.408	67 (20)	127 (17)	0.176
Moderate/substantial	256 (68)	597 (70)		261 (80)	621 (83)	
						
*Exposure in job up to 2 years before birth*						
No	119 (32)	298 (35)	0.231			
Yes	260 (69)	556 (65)				
						
*Exposure in job up to 1 year before birth*						
No	138 (36)	333 (39)	0.389	36 (11)	105 (14)	0.171
Yes	241 (64)	521 (61)		292 (90)	643 (86)	
						
*Exposure up to 1 year after birth*						
No	151 (65)	370 (69)	0.052			
Low	70 (30)	126 (23)				
Moderate/substantial	10 (4)	41 (8)				

Abbreviation: ELF=extremely low frequency.

**Table 3:** Risk of acute lymphoblastic leukaemia and exposure to ELF

		**Unadjusted mother**		**Adjusted mother**		**Unadjusted father**		**Adjusted father**	
**Exposure time period**	**Exposure**	**OR**	**95% CI**	**OR**	**95% CI**	**OR**	**95% CI**	**OR**	**95% CI**
Exposure any time before birth	Moderate/substantial	0.89	0.69–1.16	0.96	0.74–1.25	0.80	0.57–1.11	0.78	0.56–1.09
Exposure in jobs 2 years before birth	Any exposure	1.17	0.90–1.52	1.13	0.87–1.48				
Exposure in jobs 1 year before birth	Any exposure	1.12	0.87–1.43	1.11	0.86–1.44	1.32	0.88–1.98	1.33	0.88–1.99
Exposure up to 1 year after birth	Low	1.36	0.96–1.93	1.34	0.94–1.91				
	Moderate/substantial	0.60	0.29–1.22	0.61	0.29–1.25				

Abbreviations: ELF=extremely low frequency; OR=odds ratio; CI=confidence interval.

Maternal models adjusted for child sex, child age at diagnosis, socioeconomic status, maternal smoking during pregnancy, maternal drinking during pregnancy and maternal age at the time of birth. Paternal models adjusted for child sex, child age at diagnosis, socioeconomic status, paternal smoking during birth year and paternal drinking alcohol 1 year before pregnancy. Reference category for all models is no exposure.
